# Identification of key genes and biological pathways associated with vascular aging in diabetes based on bioinformatics and machine learning

**DOI:** 10.18632/aging.205870

**Published:** 2024-05-27

**Authors:** Sha Wang, Xia Wang, Jing Chen, Min Wang, Chi Zhang

**Affiliations:** 1 Department of Endocrinology, Hunan Provincial People’s Hospital, The First Affiliated Hospital of Hunan Normal University, Changsha, Hunan, China

**Keywords:** diabetes, cardiovascular disease, vascular aging, vascular smooth muscle cell (VSMC), bioinformatics, machine learning

## Abstract

Vascular aging exacerbates diabetes-associated vascular damage, a major cause of microvascular and macrovascular complications. This study aimed to elucidate key genes and pathways underlying vascular aging in diabetes using integrated bioinformatics and machine learning approaches. Gene expression datasets related to vascular smooth muscle cell (VSMC) senescence and diabetic vascular aging were analyzed. Differential expression analysis identified 428 genes associated with VSMC senescence. Functional enrichment revealed their involvement in cellular senescence, ECM-receptor interaction, PI3K-Akt and AGE-RAGE signaling pathways. Further analysis of diabetic vascular aging datasets revealed 52 differentially expressed genes, enriched in AMPK signaling, AGE-RAGE signaling, cellular senescence, and VEGF signaling pathways. Machine learning algorithms, including LASSO regression and SVM-RFE, pinpointed six key genes: TFB1M, FOXRED2, LY75, DALRD3, PI4K2B, and NDOR1. Immune cell infiltration analysis demonstrated correlations between diabetic vascular aging, the identified key genes, and infiltration levels of plasma cells, M1 macrophages, CD8+ T cells, eosinophils, and regulatory T cells. In conclusion, this study identified six pivotal genes (TFB1M, FOXRED2, LY75, DALRD3, PI4K2B, and NDOR1) closely associated with diabetic vascular aging through integrative bioinformatics and machine learning approaches. These genes are linked to alterations in the immune microenvironment during diabetic vascular aging. This study provides a reference and basis for molecular mechanism research, biomarker mining, and diagnosis and treatment evaluation of diabetes-related vascular aging.

## INTRODUCTION

Diabetes mellitus (DM) is a complex progressive metabolic disorder characterized by hyperglycemia, dyslipidemia, inflammation, and insulin resistance. Due to unhealthy lifestyles, genetic predisposition, urbanization, and aging populations, the prevalence of diabetes has been increasing globally over the past few decades. According to the 10th edition of the International Diabetes Federation's Diabetes Atlas, the estimated prevalence of diabetes among the global population aged 20-79 years was 10.5% (536.6 million people) in 2021, and it is expected to rise to 12.2% (783.2 million people) by 2045 [1]. Diabetes can lead to various complications, and cardiovascular disease (CVD) is the major complication and cause of mortality and morbidity among diabetic patients [2]. A study has shown that the risk of CVD is 2 to 4 times higher in diabetic patients compared to non-diabetic individuals [3]. CVD complications associated with diabetes can be divided into microvascular complications, such as diabetic nephropathy and retinopathy, and macrovascular complications, including diabetic cardiomyopathy, coronary artery disease, etc. [4].

Vascular aging is the structural and functional degenerative changes that occur in the cardiovascular system, which interacts with various age-related diseases and affects the threshold, progression, severity, and prognosis of diseases. Vascular aging is common in diabetic patients and is the major cause of diabetes-related CVD [5]. Vascular calcification is an important phenotype of vascular aging. Arterial calcification, as a common macrovascular complication in diabetic patients, is a crucial factor leading to atherosclerosis, amputation, renal failure, stroke, and refractory hypertension, increasing the incidence of cardiovascular events and mortality [6].

Vascular smooth muscle cells (VSMCs) are one of the major cell types that make up the vascular wall and have the ability to contract and relax, thereby regulating the diameter and blood flow of blood vessels. VSMC senescence plays an important role in diabetes. In diabetes, the hyperglycemic state increases intracellular oxidative stress and inflammation, causing protein and DNA damage in VSMCs, which accelerates VSMC senescence [7]. On the other hand, VSMC senescence leads to a decrease in vascular wall elasticity and an increase in wall thickness, which can cause vascular sclerosis and atherosclerotic plaque formation, thereby increasing the risk of cardiovascular disease [8, 9]. Therefore, preventing and treating VSMC senescence may help reduce the incidence of cardiovascular complications in diabetic patients.

Bioinformatics and machine learning techniques are commonly used to analyze gene and protein expression profiles, facilitating the discovery of molecular mechanisms underlying specific pathological changes. Based on the pathological mechanism of VSMC senescence-mediated diabetic vascular aging, this study employed bioinformatics and machine learning methods to explore the related genes and molecular mechanisms underlying diabetic vascular aging. The results of this study aim to further clarify the role of VSMC senescence in the occurrence and development of diabetic vascular aging and provide a reference and basis for the research on the molecular mechanisms, biomarker discovery, and diagnosis and treatment evaluation of diabetic vascular aging.

## METHODS

### Data download and preprocessing

Four gene expression datasets (GSE66280 and GSE171663, GSE121487 and GSE57329) were downloaded from the Gene Expression Omnibus (GEO) database (https://www.ncbi.nlm.nih.gov/geoprofiles/). GSE66280 and GSE171663 are gene expression datasets related to vascular smooth muscle cell (VSMC) senescence and were used to identify genes associated with VSMC senescence. GSE66280 is based on the GPL16570 platform (Affymetrix Mouse Gene 2.0 ST Array) and includes six normal VSMC samples and six VSMC senescence samples induced by high glucose. GSE171663 is based on the GPL18573 platform (Illumina NextSeq 500) and includes four normal VSMC samples and four stress-induced VSMC senescence samples. GSE121487 and GSE57329 targeted diabetic vasculopathy, both incorporating GPL7202 platform comprising gene expression from three normal and three diabetic mouse aorta samples each.

The limma R package was used to perform background correction, median normalization, and gene symbol conversion on the four datasets. The GSE121487 and GSE57329 datasets were merged and batch effects were removed using the “sva” package. Gene expression box plots and Uniform Manifold Approximation and Projection (UMAP) analysis were performed to verify the effectiveness of batch correction.

### VSMC senescence-related genes screening

The R package “limma” was used to perform differential expression analysis between normal VSMC samples (Control group) and senescent VSMC samples (Senescence group) in the GSE66280 and GSE171663 datasets, respectively. Genes with a threshold of “|fold change| >1.2 and *p* < 0.05” were selected as differentially expressed genes (DEGs). The common DEGs in the two datasets were defined as VSMC senescence related genes.

### Diabetic vascular aging-related genes screening

Differential analysis of gene expression was performed between normal mouse aorta samples (Control group) and diabetic mouse aorta samples (Diabetes group) in the merged GSE121487 and GSE57329 datasets related to diabetic vascular aging using the R package “limma”. DEGs were selected based on the threshold of “|fold change| >1.2 and *p* < 0.05”. By comparing the genes related to VSMC senescence, the common DEGs were identified as genes that affect diabetic vascular aging.

### Functional enrichment analysis and protein-protein interaction (PPI) analysis

Functional enrichment analysis, including KEGG (Kyoto Encyclopedia of Genes and Genomes) and Gene Ontology (GO) annotation, is a bioinformatics approach that associates specific sets of genes with known signaling pathways and biological functions to identify the signaling pathways and biological functions involved in the target genes. The GO annotation includes three categories: biological processes (BPs), cellular components (CCs), and molecular functions (MFs). In this study, we used the DAVID database (https://david.ncifcrf.gov) and Metascape dataset (http://metascape.org/gp/index.html#/main/step1) to perform KEGG and GO functional enrichment analysis for the VSMC senescence-related genes and diabetic vascular aging-related genes, respectively. For DAVID analysis, the functional annotation tool was used with the following settings: Species: Mus musculus, Annotation Categories: KEGG_PATHWAY, GO_TERM_BP_DIRECT, GO_TERM_CC_DIRECT, GO_TERM_MF_DIRECT, and the default settings for remaining parameters. For Metascape analysis, the “Express Analysis” tool was used with the following parameters: Species: Mus musculus, Analysis Mode: Custom Analysis, and the default settings for remaining parameters. A *p*-value < 0.05 was considered statistically significant in these functional enrichment analyses. In addition, to identify significantly different signaling pathways between two different biological conditions in the dataset, we chose KEGG gene sets genomes as the reference genome and performed Gene Set Enrichment Analysis (GSEA) using GSEA software version 4.0.1 and gene set variation analysis (GSVA) using the “GSVA” R package. A *p*-value < 0.05 was considered significant in these functional enrichment analysis results.

To explore the interactions among the diabetic vascular aging-related genes, we uploaded these genes to the STRING database (https://string-db.org/). A PPI network model was constructed using Cytoscape 3.7.1 software, and the topological parameters of each target were calculated.

### Selection of key genes based on machine learning

Two machine learning algorithms, two robust machine learning algorithms, Least Absolute Shrinkage and Selection Operator (LASSO) regression and Support Vector Machine-Recursive Feature Elimination (SVM-RFE), were used to select key genes related to diabetic vascular aging from the diabetic vascular aging dataset. LASSO regression, implemented using the “glmnet” R package, was first applied to the set of genes related to diabetic vascular aging. The optimal regularization strength (λ) was determined through 10-fold cross-validation, and the LASSO model was trained on the entire dataset using the selected λ value. Genes with non-zero coefficients above a threshold of |coefficient| >0.001 were considered important features. In parallel, SVM-RFE analysis was conducted using the “e1071” R package, which iteratively eliminates the least important features from the SVM model based on the absolute values of the SVM weight vector. A grid search combined with 5-fold cross-validation was performed to determine the optimal number of features to retain. The intersection genes obtained from the two analyses were considered as key genes related to vascular aging in diabetes.

### Immune infiltration analysis

CIBERSORTx (https://cibersortx.stanford.edu/) is a computational tool based on single-cell RNA sequencing (scRNA-seq) data that can be used to infer the types and relative proportions of immune cells in tissue samples. In this study, the gene expression data of each sample in the diabetic vascular aging dataset was uploaded to the CIBERSORTx database to calculate the infiltration levels of 22 immune cell types. Wilcoxon rank-sum test was used to compare the differences in immune cell infiltration between normal vascular tissue and diabetes vascular tissue, and a *p*-value < 0.05 was considered significant. Correlations were then examined between immune cells, as well as between key genes and immune cells.

### Expression of key genes and receiver operating characteristic (ROC) analysis

The Wilcoxon rank-sum test was used to analyze the expression levels of key genes in GSE66280, GSE171663, and the merged diabetic vasculopathy dataset (merged GSE121487 and GSE57329 datasets). To evaluate the specificity and sensitivity of these key genes in the diagnosis of diabetic vascular aging, we conducted receiver operating characteristic (ROC) analysis on GSE66280, GSE171663, and the merged diabetic vasculopathy dataset, and determined the area under the curve (AUC) values using the “pROC” package.

### Availability of data and materials

The entire gene expression profile data in this study were downloaded from the Gene Expression Omnibus (GEO) database (Website: https://www.ncbi.nlm.nih.gov/geo/).

## RESULTS

### Screening and functional enrichment analysis of VSMC senescence-related genes

**Figure 1:**
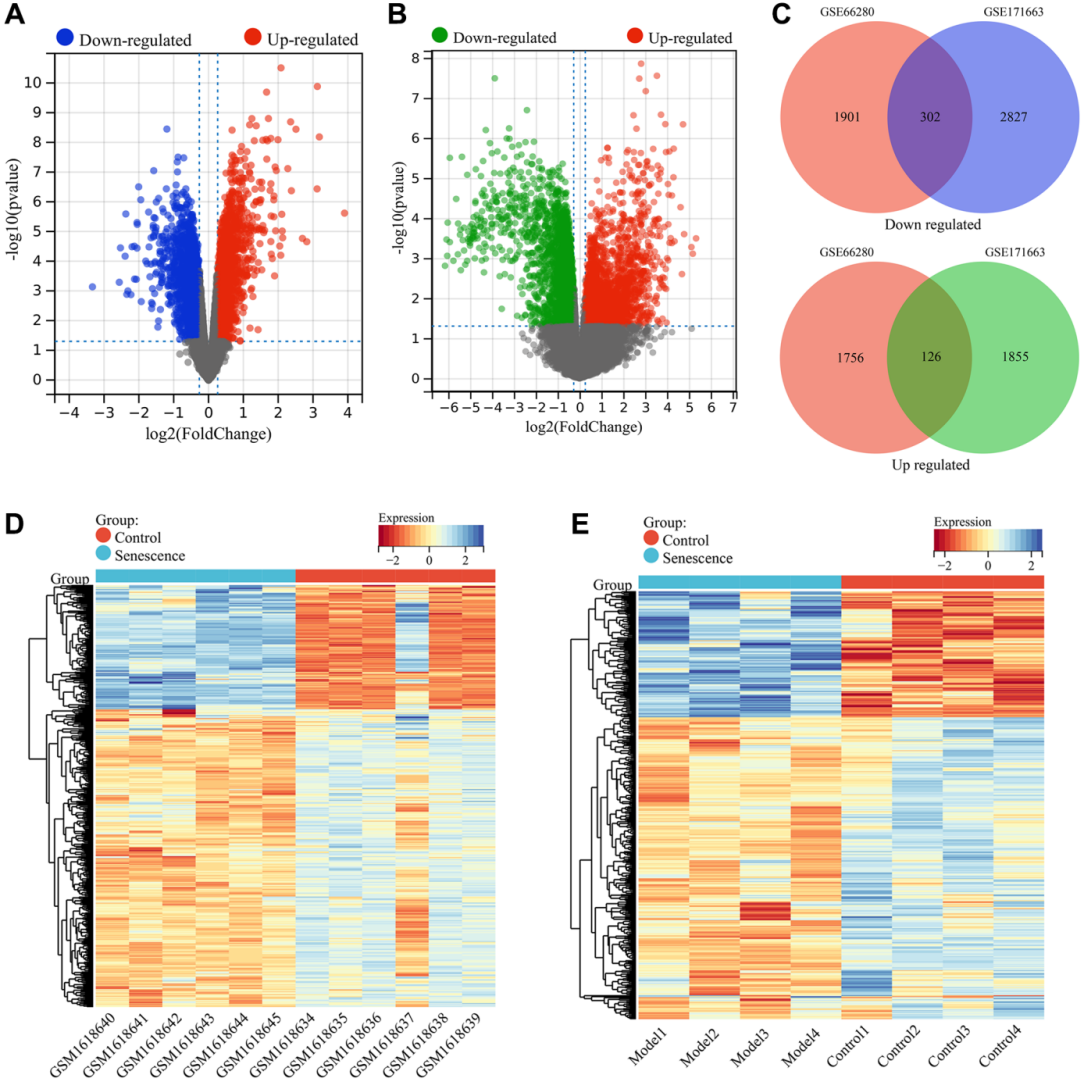
**Screening of VSMC senescence-related genes.** (**A**) Volcano plot showing differential expression analysis in the GSE66280 dataset. (**B**) Volcano plot showing differential expression analysis in the GSE171663 dataset. (**C**) Identification of commonly DEGs (VSMC senescence-related genes). (**D**) Heatmap of VSMC senescence-related genes expression in the GSE66280 dataset. (**E**) Heatmap of VSMC senescence-related genes expression in the GSE171663 dataset.

A total of 4085 DEGs were identified between senescent VSMC samples (Senescence group) and normal VSMC samples (Control group) in the GSE66280 dataset, including 1882 upregulated genes and 2203 downregulated genes in the Senescence group (Figure 1A). In the GSE171663 dataset, a total of 5110 DEGs were identified, including 1981 upregulated genes and 3129 downregulated genes (Figure 1B). Through a comparison of the two datasets, 428 common DEGs were identified, including 126 upregulated genes and 302 downregulated genes in the Senescence group (Figure 1C). These common DEGs were considered to be related to VSMC senescence. The expression of these VSMC senescence related genes in the GSE66280 and GSE171663 datasets was shown in the heat map (Figure 1D, 1E).

**Figure 2:**
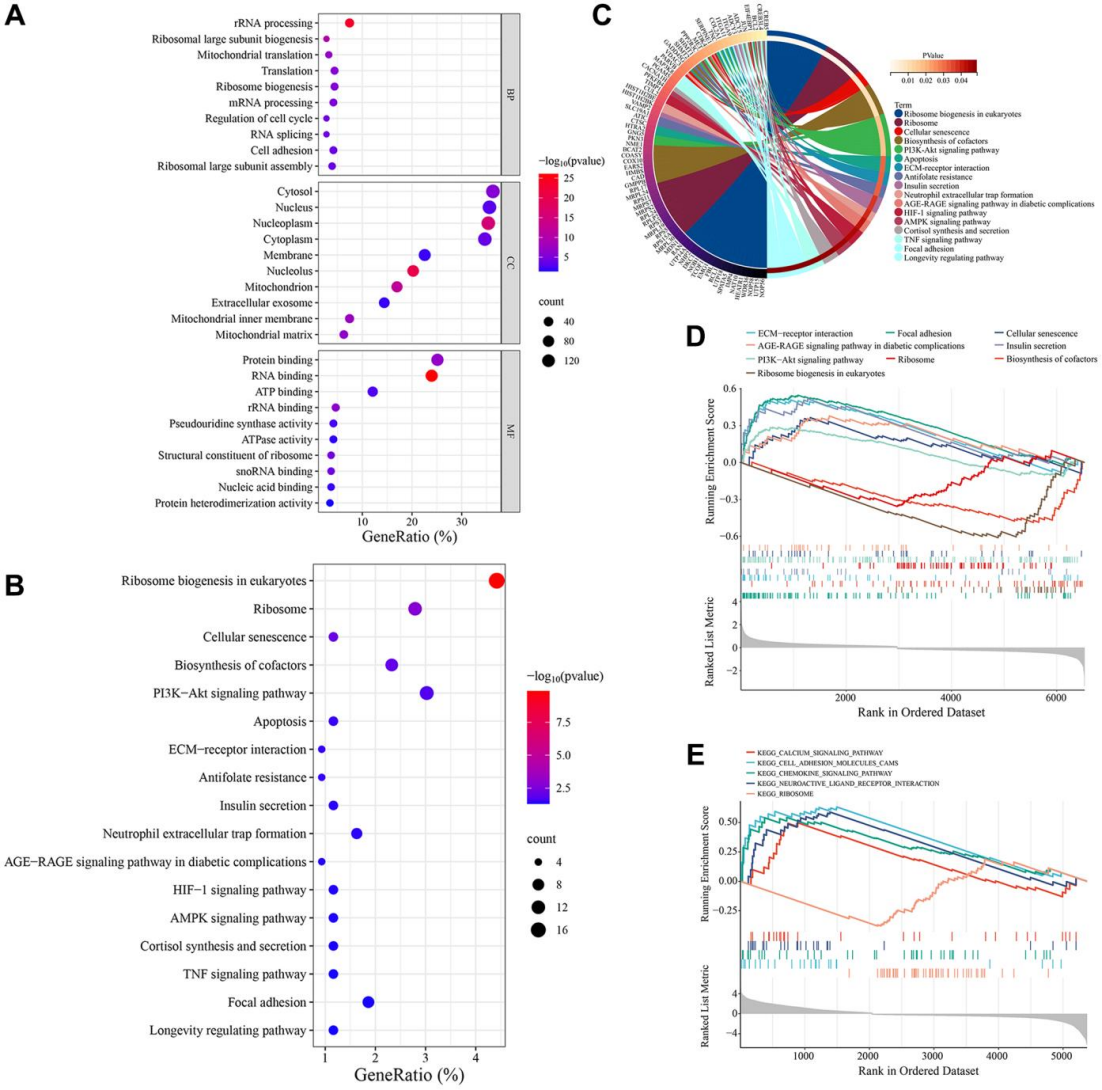
**Enrichment analysis of VSMC senescence-related genes.** (**A**) GO annotation. (**B**) KEGG pathway enrichment analysis. (**C**) The interaction of KEGG signaling pathways and their associated genes. (**D**) GSEA in the GSE66280 dataset. (**E**) GSEA in the GSE171663 dataset.

Functional enrichment analysis was performed on these common DEGs. GO annotation results showed that these genes exerted multiple functions, such as protein binding, RNA binding, ATP binding, rRNA binding, and pseudouridine synthase activity. These genes were mainly enriched in many cellular components (CCs), such as cytosol, nucleus, nucleoplasm, cytoplasm, membrane. And they were involved in several BPs such as rRNA processing, ribosomal large subunit biogenesis, mitochondrial translation, ribosome biogenesis, and regulation of cell cycle. The top 10 most significantly enriched GO terms were displayed in Figure 2A. KEGG pathway enrichment analysis showed that vascular smooth muscle cell senescence-related genes were enriched in 17 signaling pathways, including ribosome biogenesis in eukaryotes, ribosome, cellular senescence, biosynthesis of cofactors, PI3K-Akt signaling pathway (Figure 2B, 2C). We further analyzed the expression differences of these KEGG pathways between different groups in the GSE66280 and GSE171663 datasets using gene set enrichment analysis (GSEA). The results showed that in the GSE66280 dataset, 9 KEGG pathways were significantly different between normal and senescent VSMCs. Six KEGG pathways, such as cellular senescence, ECM-receptor interaction, PI3K-Akt signaling pathway, and the AGE-RAGE signaling pathway in diabetic complications, were activated in senescent VSMCs, while three KEGG pathways, including ribosome biogenesis in eukaryotes, biosynthesis of cofactors, and ribosome, were inhibited in senescent VSMCs (Figure 2D). In the GSE171663 dataset, 4 KEGG pathways, including cellular senescence, ECM-receptor interaction, focal adhesion, and apoptosis, were activated in senescent VSMCs, while the ribosome pathway was inhibited (Figure 2E).

### Screening and functional enrichment analysis of diabetic vascular aging-related genes

**Figure 3:**
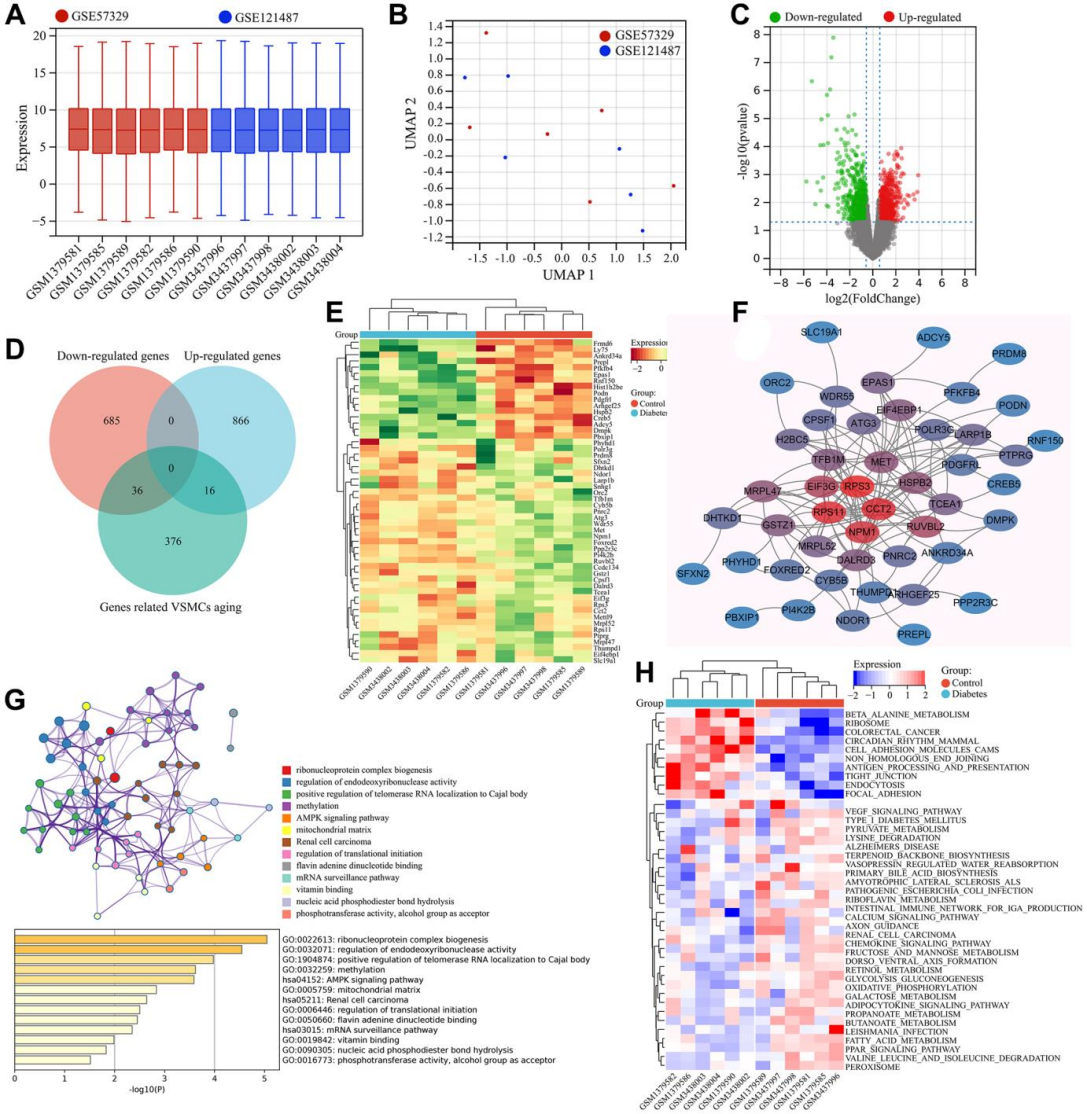
**Screening and functional enrichment analysis of diabetic vascular aging-related genes.** (**A**) Boxplots of gene expressions after the removal of inter-batch effect. (**B**) UMAP analysis after the removal of inter-batch effect. (**C**) Volcano plot of differential expression analysis. (**D**) Identification of common DEGs (diabetic vascular aging-related genes). (**E**) Heatmap of gene expression for diabetic vascular aging-related genes. (**F**) PPI analysis of diabetic vascular aging-related genes. (**G**) KEGG and GO enrichment analysis of diabetic vascular aging-related genes. (**H**) Heatmap of differentially expressed signaling pathways (GSVA).

The GSE121487 and GSE57329 expression matrices were combined and batch effects were removed. Gene expression boxplot and principal component analysis were performed on the corrected data. As shown in the gene expression boxplot, the data distribution between the different datasets became consistent after batch effect removal, with the medians aligned on a single line (Figure 3A). UMAP analysis showed that samples from GSE121487 and GSE57329 were randomly distributed, indicating that the influence of batch effect had been eliminated (Figure 3B). After batch effect removal, a total of 1604 differentially expressed genes were identified between diabetes vascular samples (Diabetes group) and normal vascular samples (Control group) (Figure 3C). By comparing with the screened VSMC senescence-related genes, 52 differentially expressed genes were obtained, including 16 significantly upregulated genes in the Diabetes group and 36 significantly downregulated genes (Figure 3D, Supplementary Table 1). These genes were considered as diabetic vascular aging-related genes. The expression of these diabetic vascular aging-related genes in the diabetes group and control group was shown in the heat map (Figure 3E).

The interactions among these diabetic vascular aging-related genes were visualized in a PPI network. Among them, CCT2, RPS11, RPS3, NPM1, EIF3G, TFB1M and DALRD3 were identified as the genes with the highest “degree” values in the PPI network, suggesting their pivotal roles in the regulation of diabetic vascular aging-related genes (Figure 3F). Functional enrichment analysis of these genes was performed using the Metascape database. The results revealed that these genes were mainly enriched in 41 GO terms and 8 KEGG signaling pathways, such as ribosomal subunit, AMPK signaling pathway, unfolded protein binding, ribonucleoprotein complex biogenesis, oxidoreductase activity, and RNA modification. To visualize the interactions, the GO terms and KEGG pathways with the strongest interconnections were selected for network analysis (Figure 3G). Furthermore, the GSVA results showed that 30 KEGG signaling pathways, such as the beta-alanine metabolism, ribosome, mammalian circadian rhythm, endocytosis and cell adhesion molecules (CAMs), were significantly upregulated in diabetic vascular aging. In contrast, 10 KEGG signaling pathways, such as fatty acid metabolism, VEGF signaling, PPAR signaling and peroxisome, were significantly downregulated in diabetes-associated vascular aging (Figure 3H).

### Selection of key genes based on machine learning

**Figure 4:**
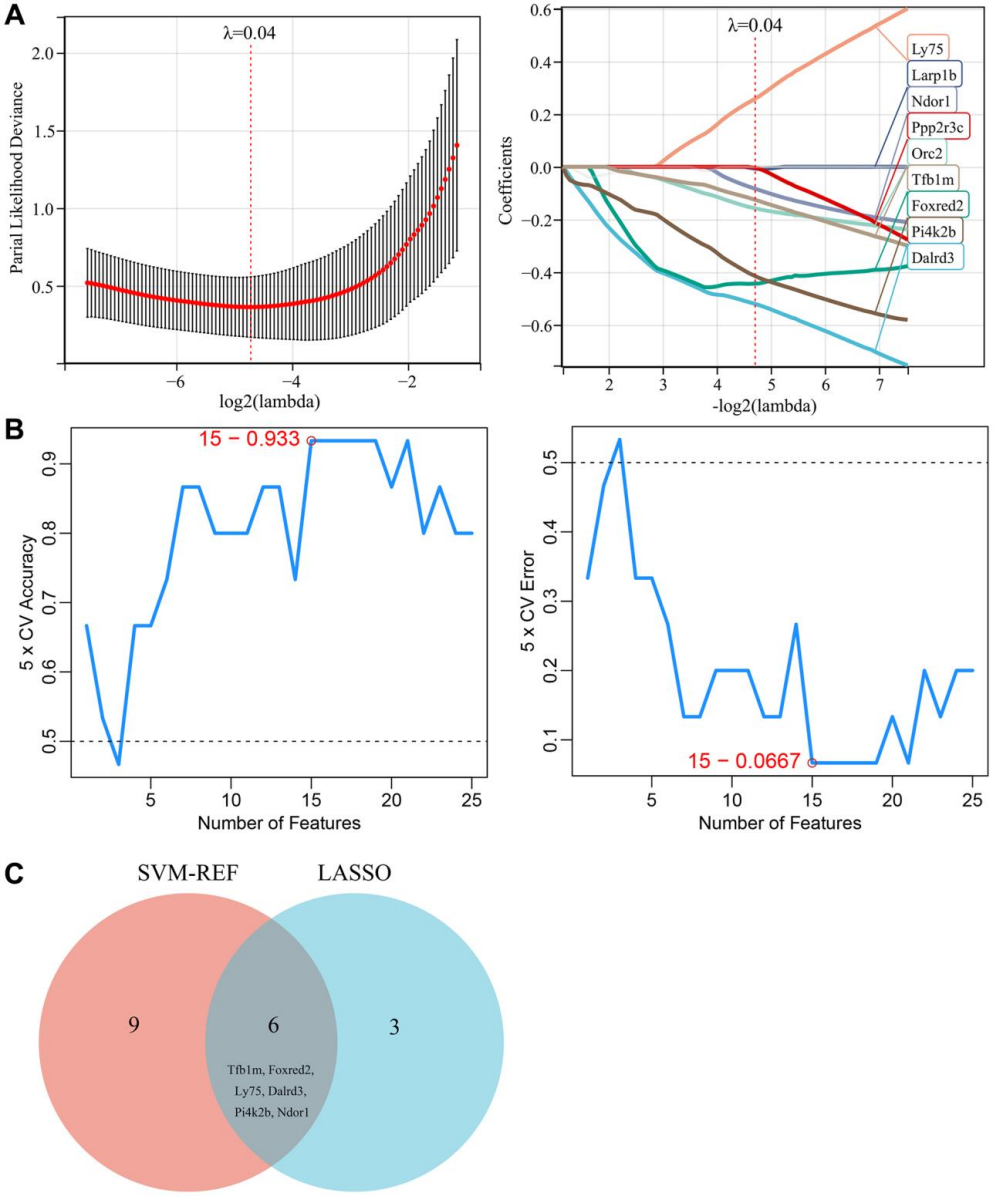
**Selection of key genes based on machine learning.** (**A**) LASSO regression model screened the candidate genes. (**B**) SVM-RFE algorithm screened the potential feature genes. (**C**) The Venn diagram showed the overlap of key genes between the above two machine learning algorithms.

Two machine learning algorithms were used in this study to further screen for key genes associated with vascular aging in diabetes. These key genes could serve as potential biomarkers or therapeutic targets for diabetes associated vascular aging. First, a LASSO regression model was designed based on diabetes vascular samples and normal vascular samples. When λ was set to 0.04, the LASSO regression model accurately distinguished between diabetes vascular samples and normal vascular samples, yielding 12 candidate genes (Figure 4A). Moreover, SVM–REF analysis was performed and identified 15 feature genes (Figure 4B). Finally, the results from the two algorithms were combined, yielding 6 key genes associated with diabetic vascular aging, including TFB1M, FOXRED2, LY75, DALRD3, PI4K2B, and NDOR1 (Figure 4C).

### Immune infiltration analysis

**Figure 5:**
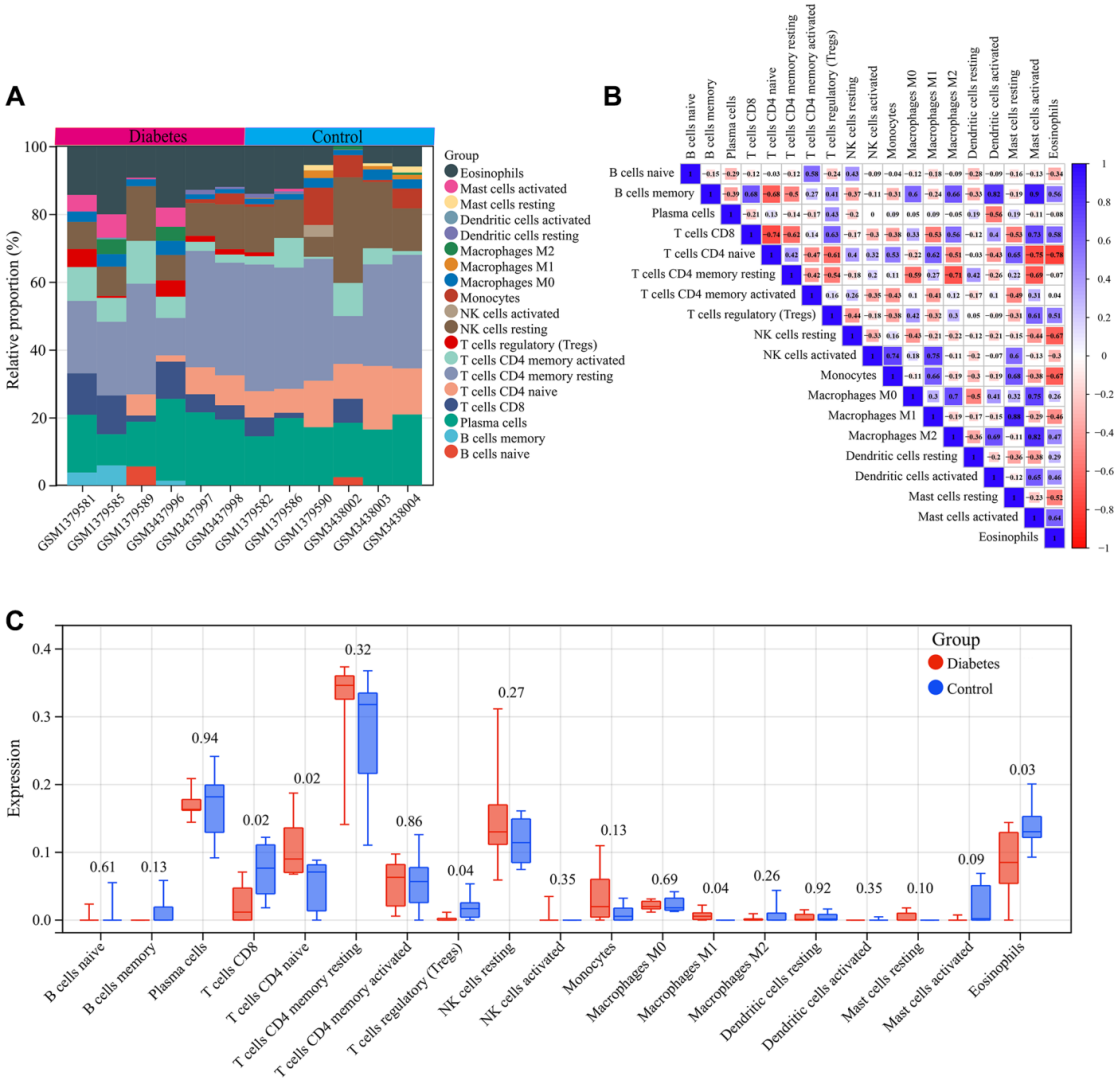
**Immune infiltration analysis.** (**A**) Bar graph of immune cell infiltration in each vascular sample. (**B**) Correlation between 19 characteristic immune cells. (**C**) Comparison of the infiltration of 19 immune cell types in the vascular samples of the diabetes and control groups.

We analyzed the immune cell infiltration in diabetic vascular tissues and normal vascular tissues. A total of 19 infiltrating immune cell types were identified in the vascular tissues. The bar graph displayed the type and number of immune cell infiltrates in each sample (Figure 5A). Subsequently, we assessed the correlation between these immune cell populations. A strong positive correlation between Mast cells activated and B cells memory (r = 0.9) and a strong negative correlation between T cells CD4 naive and Eosinophils (r = −0.78) was found (Figure 5B).

In addition, we compared the differences in infiltration levels of the 19 immune cell types between diabetic vascular tissues and normal vascular tissues. Compared to normal vascular tissues, T cells CD4 naive and Macrophages M1 showed significantly higher expression in diabetic vascular tissues, whereas T cells CD8, Eosinophils, and T cells regulatory (Tregs) exhibited significantly lower expression in diabetic vascular tissues (Figure 5C).

**Figure 6:**
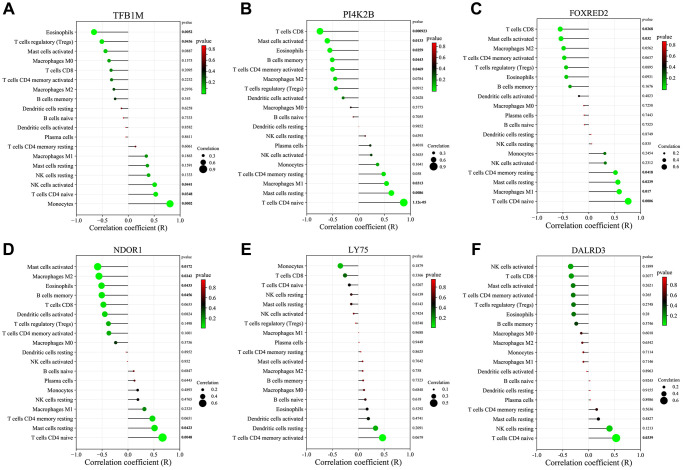
**Correlation of key genes related to diabetic vascular aging with immune cell infiltration.** (**A**) TFB1M1. (**B**) PI4K2B. (**C**) FOXRED2. (**D**) NDOR1. (**E**) LY75. (**F**) DALRD3.

Furthermore, we investigated the correlation between the expression of six key genes (TFB1M, FOXRED2, DALRD3, PI4K2B, and NDOR1) and the infiltration of the 19 immune cell types (Figure 6A–6F). The differentially expressed immune cells in diabetic vascular tissues and normal vascular tissues showed a certain correlation with the expression of these key genes. Specifically, TFB1M expression displayed a significant positive association with the infiltration status of T cells CD4 naive and negatively correlated with the infiltration status of Eosinophils and T cells regulatory (Tregs). PI4K2B expression showed a significant positive link to the infiltration status of T cells CD4 naive and Macrophages M1 and negatively correlated with T cells CD8 and Eosinophils. FOXRED2 expression was significantly positively linked to the infiltration status of T cells CD4 naive and Macrophages M1 and negative correlation with the infiltration status of T cells CD8. NDOR1 expression exhibited a significantly positive correlation with naive CD4 T cell infiltration levels and a significantly negative correlation with Eosinophil infiltration levels. DALRD3 expression showed a significant positive correlation with T cells CD4 naive infiltration levels. No significant correlation was found between LY75 and immune cell infiltration.

### Expression of key genes and ROC analysis

**Figure 7:**
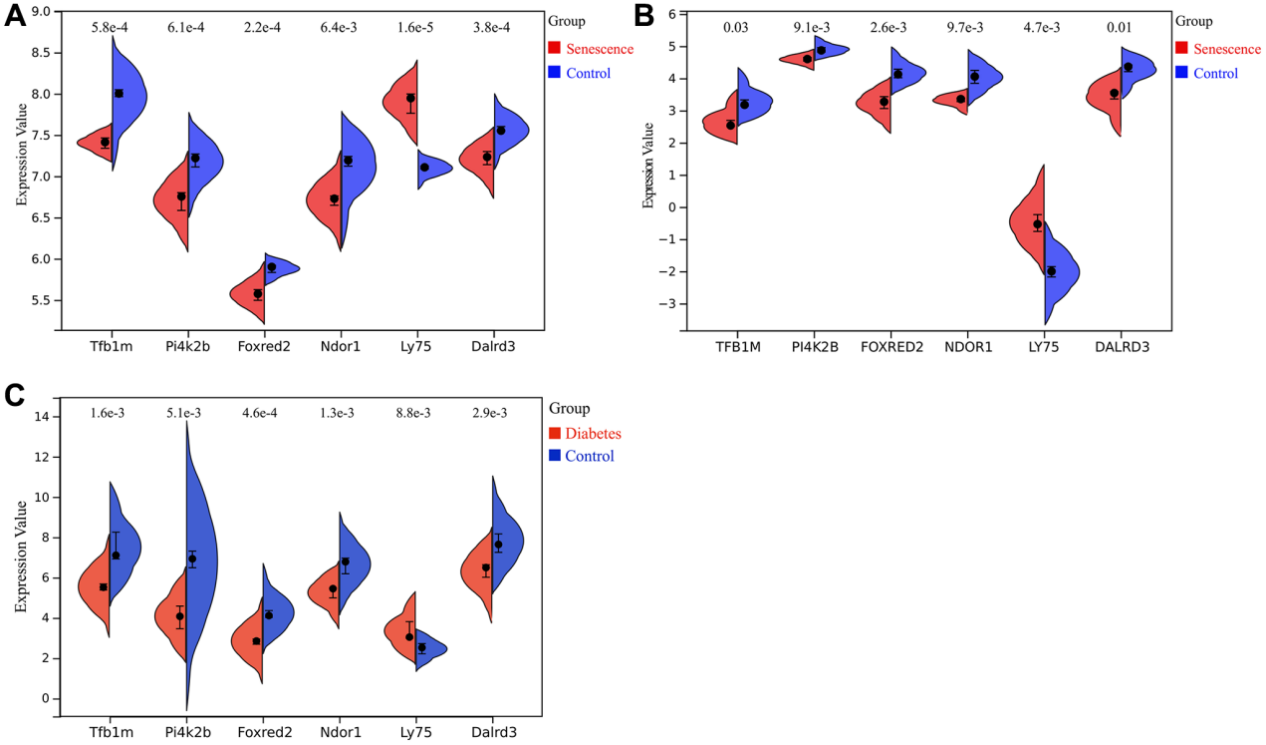
**Expression of key genes related to diabetic vascular aging.** (**A**) Expression of Tfb1m1, Pi4k2b, Foxred2, Ndor1, Ly75 and Dalrd3 in GSE66280 dataset. (**B**) Expression of TFB1M1, PI4K2B, FOXRED2, NDOR1, LY75, DALRD3 in GSE171663 dataset. (**C**) Expression of Tfb1m1, Pi4k2b, Foxred2, Ndor1, Ly75 and Dalrd3 in the merged diabetic vasculopathy dataset (merged GSE121487 and GSE57329 datasets).

In datasets GSE66280 (Figure 7A) and GSE171663 (Figure 7B), the expression of key genes TFB1M, FOXRED2, DALRD3, PI4K2B, and NDOR1 was significantly downregulated in senescent VSMCs compared to normal VSMCs, while the expression of LY75 gene was significantly upregulated. Similarly, in the merged diabetic vasculopathy dataset (GSE121487 and GSE57329), TFB1M, FOXRED2, DALRD3, PI4K2B and NDOR1 were markedly downregulated, whereas LY75 was upregulated in diabetic blood vessels (Figure 7C).

**Figure 8:**
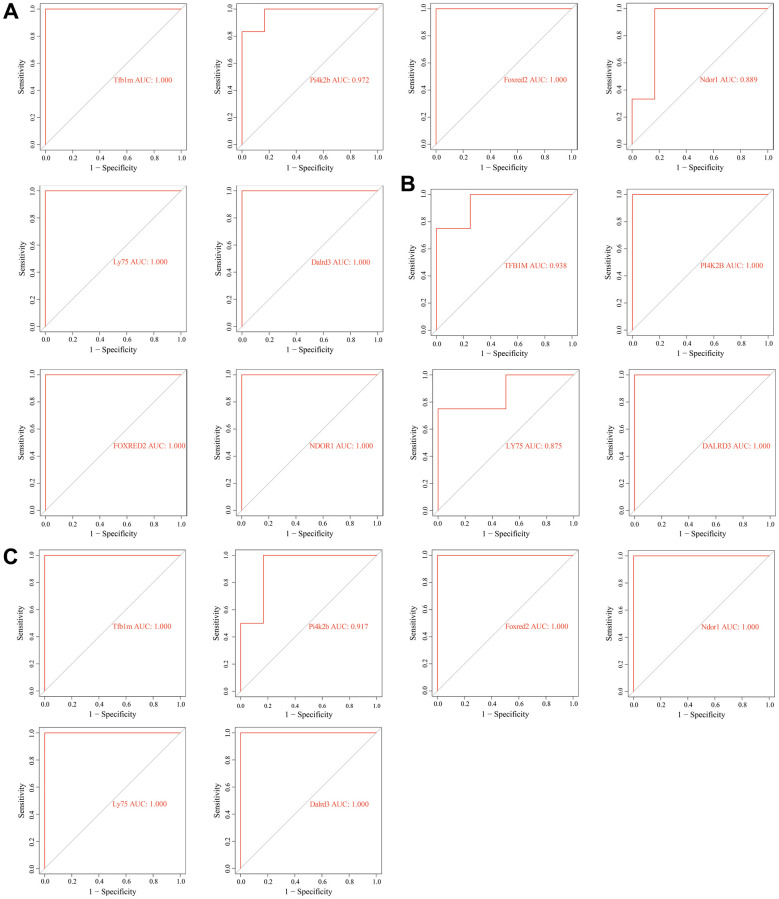
**Receiver operating characteristic (ROC) analysis of key genes related to diabetic vascular aging.** (**A**) ROC curves of Tfb1m1, Pi4k2b, Foxred2, Ndor1, Ly75 and Dalrd3 in GSE66280 dataset. (**B**) ROC curves of TFB1M1, PI4K2B, FOXRED2, NDOR1, LY75, DALRD3 in GSE171663 dataset. (**C**) ROC curves of Tfb1m1, Pi4k2b, Foxred2, Ndor1, Ly75 and Dalrd3 in the merged diabetic vasculopathy dataset (merged GSE121487 and GSE57329 datasets).

The ROC analysis revealed that these key genes can accurately distinguish between senescent VSMCs and normal VSMCs, as well as between normal vascular samples and diabetic vascular aging samples. The AUC values of TFB1M, FOXRED2, LY75, DALRD3, PI4K2B, and NDOR1 were all greater than 0.8 across all analyzed datasets, indicating their strong potential as biomarkers for vascular aging (Figure 8A–8C).

## DISCUSSION

Diabetes mellitus is a complex and progressive metabolic disorder characterized by hyperglycemia that accelerates vascular aging. Vascular aging exacerbates diabetes-associated vascular damage, particularly in highly vascularized organs such as the kidneys, heart, and brain, which are the main causes of various microvascular and macrovascular complications [8]. Inhibiting vascular aging is of great significance to prevent the occurrence of various cardiovascular complications associated with diabetes. VSMCs are one of the main components of vascular walls and play an irreplaceable role in maintaining the stable structure and function of blood vessels. Vascular aging is manifested at the cellular level as morphological abnormalities of VSMCs, and histologically as an increase in collagen, a decrease in elastic fibers, fracture, and more importantly arteriosclerosis and calcification [10]. At the molecular level, vascular aging in diabetes is associated with high glucose-induced VSMC senescence, telomere erosion, and persistent DNA damage [5, 11, 12]. Bartoli-Leonard et al. have shown that SIRT1 deficiency in diabetic patients can induce VSMC senescence and DNA damage, thus accelerating vascular aging [5]. Milk fat globule–epidermal growth factor 8 (MFG-E8), a glycoprotein expressed in VSMCs, is highly expressed in aged arteries and is considered a useful biomarker of arterial aging. Yu et al. demonstrated that MFG-E8 promotes arterial injury in diabetic mice through activation of the extracellular signal-regulated kinase (ERK) and monocyte chemoattractant protein-1 (MCP-1) signaling pathways [13]. Chiang et al. found that MFG-E8 promotes vascular aging by regulating the TGF-β1 signaling pathway, leading to VSMC osteogenic differentiation and biomineralization [14]. Although some genes have been shown to be associated with diabetes-related vascular aging, the specific molecular mechanisms of diabetes-related vascular aging still need further research.

In this study, we identified several signaling pathways that are closely related to vascular aging in diabetes, such as the AMPK signaling pathway, AGE-RAGE signaling pathway, cellular senescence, and VEGF signaling pathway. AMPK (AMP-activated protein kinase) is an energy-sensitive protein kinase that can be activated by changes in the AMP/ATP ratio within the cell to regulate energy metabolism, lipid metabolism, cell proliferation, and autophagy among other biological processes. AMPK signaling pathway is suppressed in diabetes and its various cardiovascular complications, activating the AMPK signaling pathway has beneficial effects in improving diabetes and its related cardiovascular complications [15, 16]. Phadwal found that metformin could improve VSMC senescence and alleviate vascular aging in diabetes by enhancing cell autophagy mediated by the AMPK signaling pathway [17]. AGE-RAGE (Advanced Glycation End Products-Receptor for Advanced Glycation End Products) signaling pathway is an important pathological mechanism of vascular complications in diabetes. AGEs are a class of highly converted products generated by the reaction of reducing sugars with amino compounds. They can cause abnormal cell function and damage through signal transduction mediated by the RAGE receptor. In diabetes patients, high blood sugar leads to increased generation of AGEs, which then activate the RAGE receptor, inducing cell senescence, intracellular inflammatory reactions, and oxidative stress, accelerating the process of vascular aging [18, 19]. VEGF (vascular endothelial growth factor) is a critical regulatory factor for VSMC growth and angiogenesis. In diabetic patients, elevated blood glucose and AGEs levels inhibit the normal function of the VEGF signaling pathway [20, 21]. Activation of the VEGF signaling pathway can promote the proliferation and migration of VSMCs, prevent cell senescence, and thereby facilitate repair and reconstruction of the vascular wall [22, 23].

We used machine learning to identify six key genes closely associated with vascular aging in diabetes: TFB1M, FOXRED2, LY75, DALRD3, PI4K2B, and NDOR1. The expression of TFB1M, FOXRED2, DALRD3, PI4K2B, and NDOR1 was negatively correlated with vascular aging in diabetes, while LY75 expression was positively correlated. Some studies have suggested potential links between these genes and vascular aging in diabetes. TFB1M (Transcription factor B1 mitochondrial) is a transcription factor that is expressed in mitochondria and is involved in mitochondrial DNA transcription and repair. Variations or low expression of TFB1M have been associated with decreased insulin secretion in type 2 diabetes patients [24]. As a mitochondrial transcription factor, TFB1M can regulate mitochondrial DNA transcription and repair, thereby protecting mitochondrial function and improving energy metabolism and insulin secretion in diabetes [25]. Moreover, low expression of TFB1M has also been associated with cellular senescence and organelle damage in diabetes [26]. However, no studies have yet shown whether low expression of TFB1M in diabetes can cause vascular aging. FOXRED2 (FAD-dependent oxidoreductase domain-containing protein 2) is a novel endoplasmic reticulum (ER) resident protein, downregulation of which can lead to ER stress in cells [27]. ER stress is a mechanism that leads to VSMC senescence [28]. However, there is currently no direct evidence of the effect of FOXRED2 on vascular aging in diabetes. LY75 (Lymphocyte antigen 75, also known as DEC205), is an important antigen-presenting molecule in the immune system. Studies have shown that LY75 is a susceptibility gene for various cardiovascular and metabolic diseases [29, 30]. DALRD3 (DALR anticodon-binding domain-containing protein 3) is a protein that facilitates the formation of the 3-methylcytosine (m3C) modification at position 32 of specific arginine tRNA isoacceptors. The m3C modification plays a crucial role in the proper folding, stability, and function of tRNAs, which are essential for accurate protein translation [31]. Dysregulation of DALRD3 and subsequent loss of m3C modification in arginine tRNAs have been implicated in severe neurological disorders, such as epileptic encephalopathy [31]. In the context of diabetic vascular aging, downregulation of DALRD3 may impair the maturation and function of arginine tRNAs, leading to aberrant protein synthesis and potentially contributing to vascular dysfunction. However, further studies are needed to elucidate the specific mechanisms by which DALRD3 dysregulation and the consequent loss of m3C modification in arginine tRNAs contribute to the pathogenesis of vascular aging in diabetes. PI4K2B (Phosphatidylinositol 4-kinase type 2-beta) is a lipid kinase that regulates membrane trafficking and signaling pathways [32]. Dysregulation of PI4K2B could disrupt important cellular processes, such as vesicle transport and signal transduction, contributing to vascular dysfunction in diabetes. NDOR1 (NADPH-dependent diflavin oxidoreductase 1) is a NADPH oxidoreductase that participates in cellular redox regulation [33]. Downregulation of NDOR1 in diabetic vasculature could impair antioxidant defense mechanisms, leading to increased oxidative stress and accelerated vascular aging. In summary, our study identified six key genes associated with vascular aging in diabetes, which may become biomarkers and therapeutic targets for this condition.

In addition, immune factors are also important contributors to vascular senescence in diabetes. In diabetes, the levels of inflammatory cytokines are significantly elevated, leading to VSMC damage and thickening of the vascular wall, resulting in vascular aging [34, 35]. Abnormal activation and regulation of immune cells are also associated with vascular aging in diabetes [36]. For example, T cells, particularly CD4+ helper T cells and CD8+ cytotoxic T cells, are key mediators of vascular inflammation in diabetes. Upon activation, these T cells release pro-inflammatory cytokines (e.g., IFN-γ, TNF-α) and induce the recruitment and activation of other immune cells, perpetuating the inflammatory cascade [37, 38]. Activated T cells can also directly interact with smooth muscle cells, leading to vascular remodeling and stiffening [39]. Activated M1 macrophages secrete a plethora of inflammatory mediators, including IL-1β, IL-6, and reactive oxygen species, which can directly damage vascular cells and promote VSMC senescence [40]. In the present study, we found for the first time that diabetic vascular aging may be associated with the level of infiltration of immune cells such as T cells CD4 naive, Macrophages M1, T cells CD8, Eosinophils and T cells regulatory (Tregs) in vascular tissue. The key genes of diabetic vascular aging, TFB1M, FOXRED2, DALRD3, PI4K2B and NDOR1, are also potentially linked to the level of infiltration of these immune cells.

Our study provides valuable insights into the molecular mechanisms underlying diabetic vascular aging and identifies potential key genes and pathways through comprehensive bioinformatics analyses and machine learning approaches. However, it is essential to acknowledge certain limitations. The datasets were derived from mouse models, which may not fully recapitulate the complexities of human diabetic vascular aging, highlighting the need for validation in human samples. Additionally, the machine learning algorithms employed, although robust, may be subject to biases arising from algorithm assumptions and training data characteristics, potentially overlooking important genes or pathways. Future research should aim to validate the identified key genes and pathways through functional experiments, and integrate multi-omics data sources for a more comprehensive understanding. Addressing these limitations and continuing to explore this complex and clinically significant area will be crucial for advancing our knowledge and developing effective interventions for diabetic vascular aging.

## CONCLUSION

In summary, this study further clarifies the role of VSMC senescence in the development of vascular aging in diabetes. Using bioinformatics and machine learning techniques, we identified six key genes that are closely associated with vascular aging in diabetes: TFB1M, FOXRED2, LY75, DALRD3, PI4K2B, and NDOR1. These genes also have potential connections with changes in the immune microenvironment in vascular aging in diabetes. The results of this study will provide reference and basis for molecular mechanism research, biomarker exploration, and diagnosis and treatment evaluation of vascular aging in diabetes. However, the specific mechanisms of these six genes in the development of diabetic vascular aging still need to be further investigated.

## Supplementary Materials

Supplementary Table 1
