# Supplementary material for: Identification of key genes and biological pathways associated with vascular aging in diabetes based on bioinformatics and machine learning
Source: Aging (Albany NY). 2024 May 27;16(11):9369–85. doi: 10.18632/aging.205870 (PMC11210242; doi:10.18632/aging.205870)
Supplement: Supplementary Table 1 [file aging-16-205870-s001.pdf]

SUPPLEMENTARY TABLE

Supplemental Table 1. Identification of 31 diabetic vascular aging-related genes.

Rnf150	Ndor1	Mettl9	Arhgef25
Pfkfb4	Pi4k2b	Cpsf1	Ankrd34a
Hist1h2be	Tcea1	Rps11	Met
Creb5	Npm1	Eif3g	Dhtkd1
Ly75	Eif4ebp1	Phyhd1	Ruvbl2
Epas1	Dalrd3	Snhg1	Atg3
Pbxip1	Pnrc2	Orc2	Mrpl47
Prepl	Cyb5b	Ppp2r3c	Cct2
Frmd6	Slc19a1	Foxred2	Sfxn2
Adcy5	Ccdc134	Tfb1m	Gstz1
Pdgfrl	Wdr55	Ptprg	Hspb2
Podn	Larp1b	Mrpl52	Prdm8
Dmpk	Polr3g	Rps3	Thumpd1
